# What factors affect patients’ access to healthcare? Protocol for an overview of systematic reviews

**DOI:** 10.1186/s13643-020-1278-z

**Published:** 2020-01-23

**Authors:** Bryony Dawkins, Charlotte Renwick, Tim Ensor, Bethany Shinkins, David Jayne, David Meads

**Affiliations:** 1 Academic Unit of Health Economics, Leeds Institute of Health Sciences, University of Leeds, Worsley Building, Clarendon Way, Leeds, UK; 2 Asthma UK, 18 Mansell Street, London, UK; 3 Nuffield Centre for International Health and Development, Leeds Institute of Health Sciences, University of Leeds, Leeds, UK; 4 Leeds Institute of Medical Research, University of Leeds, Leeds, UK

**Keywords:** Systematic review of systematic reviews, Overview, Access to healthcare, Inequalities in access to healthcare

## Abstract

**Background:**

The importance of access to healthcare for all is internationally recognised as a global goal, high on the global agenda. Yet inequalities in health exist within and between countries which are exacerbated by inequalities in access to healthcare. In order to address these inequalities, we need to better understand what drives them. While there exists a wealth of research on access to healthcare in different countries and contexts, and for different patient groups, to date no attempt has been made to bring this evidence together through a global lens. This study aims to address that gap by bringing together evidence of what factors affect patients’ access to healthcare and exploring how those factors vary in different countries and contexts around the world.

**Methods:**

An overview of reviews will be conducted using a comprehensive search strategy to search four databases: Medline, Embase, Global Health and Cochrane Systematic Reviews. Additional searches will be conducted on the Gates Foundation, the World Health Organisation (WHO) and World Bank websites. Titles and abstracts will be screened against the eligibility criteria and full-text articles will be obtained for all records that meet the inclusion criteria or where there is uncertainty around eligibility. A data extraction table will be developed during the review process and will be piloted and refined before full data extraction commences. Methodological quality/risk of bias will be assessed for each included study using the AMSTAR 2 tool. The quality assessment will be used to inform the narrative synthesis, but a low-quality score will not necessarily lead to study exclusion.

**Discussion:**

Factors affecting patients’ ability to access healthcare will be identified and analysed according to different country and context characteristics to shed light on the importance of different factors in different settings. Results will be interpreted accounting for the usual challenges associated with conducting such reviews. The results may guide future research in this area and contribute to priority setting for development initiatives aimed at ensuring equitable access to healthcare for all.

**Systematic review registration:**

PROSPERO CRD42019144775

## Background

The Universal Healthcare Movement and Sustainable Development Goals (especially SDG3 ‘Good health and wellbeing’ and SDG10 ‘reduced inequalities’) demonstrate the importance of access to healthcare for all as a global goal, high on the global agenda [1]. Yet inequalities in health persist between and within countries which are exacerbated by inequalities in patients’ ability to access healthcare [2–4]. In order to address these inequalities and move towards these global goals that encourage fair access to safe, affordable healthcare, we need to better understand what drives these inequalities within and between countries.

The importance of access to healthcare is not a new topic and there is a wealth of research from different countries and contexts, and relating to a range of patient groups. However, to date, there has been no attempt to bring this information together, through a global lens, to examine the variations in factors that affect patients’ ability to access healthcare depending on the country, context and related cultural and social characteristics. This represents a missed opportunity to develop shared learning about what has and has not worked to improve access to healthcare. For example, shared learning on successes and failures could help systems with poor access to healthcare to learn from systems that have improved access, perhaps despite the low resource base (or other contextual factors) they may have in common. This study aims to address that gap by bringing together evidence of what factors affect patients’ access to healthcare and exploring how those factors vary and also what is common across countries and contexts around the world.

Access to healthcare has several dimensions [5]. Service availability is one dimension which is concerned with the supply of healthcare, i.e. the availability of healthcare professionals and medicines. While service availability is important to enable access, just because services are available it does not necessarily follow that they are accessible and there are a range of factors that can restrict access. These are often demand-side (related to the patient) or organisational factors. These supply- and demand-side factors are the main focus of this review.

### Research question

What factors affect patients’ access to healthcare and how do they vary in different countries and contexts around the world?

### Aim, design and setting

This study aims to identify what factors act as facilitators or barriers to patients’ ability to access healthcare, to develop understanding of what are the most important factors in different countries and contexts, and to examine the variation in these factors and the magnitude of their impact. As such, the review is not limited to one particular setting, but instead aims to consider the evidence from a global perspective to identify trends that may be useful to healthcare and development practitioners as they consider what are the best strategies and areas to target in order to move towards the global goals. Given the global perspective required to answer this research question, the size of the body of primary evidence is too large to be manageable in a conventional review. In addition, much of the primary evidence has already been compiled within systematic reviews which examine factors affecting access to healthcare within a limited region and/or within a particular clinical area. Consequently, this study has been designed as an overview of systematic reviews in order to answer the research question in a practical and manageable way [6].

## Methods/design

### Protocol and registration

This protocol is being reported in accordance with the PRISMA-P statement [7, 8] (see PRISMA-P checklist in Additional file 1). This overview protocol was registered with the International Prospective Register of Systematic Reviews (PROSPERO), registration number CRD42019144775.

### Eligibility criteria

Inclusion and exclusion criteria are summarised in Table 1. Studies will be selected for inclusion in the review based on the following criteria.

**Table 1:** Inclusion and exclusion criteria

Include	Exclude
Type of study	
Systematic reviews. Defined for inclusivity as a review in which systematic searches are conducted in at least 2 sources, at least 1 being an electronic database, and in which systematic methods are used for data extraction and synthesis.	Any study that is not a systematic review as per the definition opposite.
	Overviews (systematic reviews of systematic reviews), as the reviews they include tend to be more dated. However, reference lists will be reviewed to ensure eligible reviews they identify are included.
Subject of study	
Studies for which access to healthcare is the main focus.	Studies for which access to healthcare is not the main focus.
Systematic reviews for which barriers/facilitators to healthcare access are identified as an outcome of interest.	Studies which discuss barriers/facilitators to access only in the context of the topic but not as an outcome of the review.
	Studies which do not identify barriers or facilitators to accessing healthcare as an outcome of interest
Studies that identify demand-side (patient factors) or supply-side (healthcare provider factors) barriers/facilitators to healthcare access.	Studies that present barriers/facilitators to the implementation of a specific intervention without any focus on how that affects access to healthcare.
	Systematic reviews which focus on only one barrier, facilitator or intervention affecting access to healthcare.
Studies where barriers/facilitators to utilization are identified from the review (in this case utilization may be a proxy measure for access).	Studies that focus on ways to reduce utilization of healthcare (rather than improve access).
Types of intervention	
Any intervention or service which is a means of providing healthcare to living humans.	Interventions relating to post-mortem activities.
Types of participants	
Reviews based on any disease or condition affecting living humans.	Animal studies or post-mortem studies.
Type of publication	
Published, peer reviewed articles or non-peer reviewed reports published through Gates, WHO or World Bank websites.	Conference proceedings, protocol papers and any publication for which sufficient details to determine eligibility cannot be obtained.
Articles written in English	Articles written in any language other than English.
Time frame	
Articles published in the last 5 years, i.e. 1 January 14–current.	Articles published before 1 January 2014.

Type of study. Only systematic reviews will be included in this review, all other study designs will be excluded. For the purposes of this review, we define a systematic review as a review in which systematic searches are conducted in at least 2 sources, at least 1 being an electronic database, and in which systematic methods are used for data extraction and synthesis. This is intentionally a fairly broad and inclusive definition as we anticipate that a more stringent definition would lead to the exclusion of research that although may not be of the finest, Cochrane style, quality, can still provide useful evidence for our research question. Overviews of systematic reviews will be excluded as although this is a specific type of systematic review, the reviews they include tend to be more dated and, in addition, the primary data has already been through two levels of aggregation meaning the level of detail and nuances in the data are likely to be reduced.

Subject of the study. To be included in this review access to healthcare must be the main focus of the study and barriers and facilitators to healthcare access must be identified as an outcome of interest. Given the purpose of this review is to identify factors affecting access to healthcare and the variations in those factors in different settings, it is not possible to say what factors will be relevant or should be prioritised a priori. We will collect data on all factors reported to affect access to healthcare as presented in included articles. However, for the purposes of clarity for the reader, we might expect some barriers or facilitators to include social or cultural influences on the decision to seek healthcare, influences on the ability to travel to a place where healthcare can be provided or availability of appropriate care. In other words, studies that identify demand-side (patient factors) or supply-side (healthcare provider factors) barriers/facilitators to healthcare access can be included. Studies which focus on only one barrier, facilitator or intervention affecting access to healthcare will be excluded as these studies fail to give a complete overview of the factors affecting patients in their study setting. In addition, studies that focus on ways to reduce utilization of healthcare (rather than improve access) will be excluded. This type of study tends to focus on minimally regulated healthcare markets (like USA) and are aimed at reducing access to unnecessary care rather than increasing access of care that is needed—i.e. the purpose of this type of study is not relevant to the research question for this overview.

Types of intervention. The focus of this review is access to healthcare. A range of interventions or services can be deemed healthcare and consequently studies which focus on any intervention or service which is a means of providing healthcare to living humans is an acceptable for inclusion in this review.

Types of participants. Reviews based on any disease or condition affecting living humans can be included.

Types of publication. Published, peer reviewed articles or non-peer reviewed reports published through the Gates Foundation, WHO or World Bank websites and written in English can be included. Conference proceedings, protocol papers, and any publication for which sufficient details to determine eligibility cannot be obtained will be excluded. Due to resource constraints to conduct this research, it is not possible to include articles written in any language other than English. Unpublished reviews will not be included due to the practical difficulties in acquiring the necessary data for these studies.

Time frame. Only articles published in the last 5 years will be included. This restriction links to the overview design of the review. This overview aims to present currently relevant information and given that systematic reviews synthesise already published studies, the restriction seems appropriate to minimise more dated information. In addition, it is hoped that the restriction on publication date will minimise the bias associated with articles being included in more than one review, as repeat reviews usually occur less frequently than this. The 5-year restriction is essentially arbitrary but is expected to be more effective in minimising this type of bias than say a 10-year period when repeat reviews may be more common.

### Information sources and search methods

A comprehensive search strategy using subject headings and keywords will be used to search for potentially eligible systematic reviews in four databases: Medline, Embase, Global Health and Cochrane Systematic Reviews (from 2014 onwards). A sample search strategy is included as Additional file 2. The final search strategy will be developed with advice from information specialists by an iterative process, adapted for each database and peer reviewed to minimise errors.

Additional searches will be conducted on the Gates Foundation, the World Health Organisation (WHO) and World Bank websites.

### Screening and selection procedure

Records will be stored and managed using reference management software (Endnote and Zotero).

Titles and abstracts will be screened against the eligibility criteria and full-text articles will be obtained for all titles that meet the inclusion criteria or where there is uncertainty around eligibility. Initial screening of all articles and full-text review of potentially eligible articles will be conducted by BD. A second reviewer will independently screen 20% of all titles and abstracts and 20% of included full texts. Any discrepancies in the inclusion of abstracts or full-text articles will be resolved by discussion and reaching a consensus. In the event a consensus cannot be reached, a third author will arbitrate. Any inconsistencies in the screening approach of the independent screening will be addressed, a strategy to ensure consistency will be developed and then implemented in a repeat of the exercise.

### Data extraction

A data extraction table will be developed during the review process and will be piloted and refined before full data extraction commences. The main data to be extracted will be related to the factors identified as influencing patients’ access to healthcare, e.g. barriers and facilitators to access. These data will be collected and categorised according to the three delays model during data extraction to identify where in the patient pathway barriers and facilitators to accessing care occur in different settings and for different patient groups [9]. Due to the nature of the research question, it is not possible to make any assumptions about what these factors will be without introducing bias into the review and so data will be extracted as it is presented in the included systematic review. In addition to the factors affecting access, other data to be extracted will include: characteristics of the included reviews such as authors, year of publication, focus of the study/review question and clinical area, and features of the study design such as sources searched, limits, and eligibility criteria; the setting of the study including country/ies studied and income classification of study setting; methods used to identify factors affecting access to care; and data on included studies such as number included and types of study, e.g. quantitative or qualitative. Data on the primary articles included in reviews will not be collected systematically for all reviews as it is intended that data will be extracted from the systematic reviews not the primary articles they refer to. BD will perform data extraction for all articles included in the review and a second reviewer will confirm this data for 20% of included studies. Any discrepancies in data extraction will be resolved by discussion to reach a consensus. In the event a consensus cannot be reached, a third reviewer will arbitrate. Any inconsistencies in data extraction will be noted and a strategy to ensure consistency for the remaining data extraction will be implemented.

### Quality assessment and risk of bias

Methodological quality/risk of bias will be assessed for each included study using the AMSTAR 2 tool [10]. AMSTAR 2 is a comprehensive tool aimed at assessing the methodological quality of systematic reviews and also has several dimensions to record assessments of meta-biases within the review. The quality assessment will be used to inform the narrative synthesis, but a low-quality score will not necessarily lead to study exclusion. BD will conduct the quality assessment for each included review, of which 20% will be confirmed by a second reviewer. Any discrepancies will be resolved through discussion to reach a consensus and, in the event a consensus cannot be reached, a third reviewer will arbitrate. Any inconsistencies in the quality assessment will be noted and a strategy to ensure consistency for the remaining will be implemented. The results of the quality appraisal using the AMSTAR 2 tool will be reported alongside the findings of the overview.

Publication bias within this overview will be assessed by searching the PROSPERO database for relevant reviews that have been registered but not published. Although there is no perfect way to assess publication bias this could give an idea of other relevant research that is either ongoing or has been abandoned, and so is not included in this review.

### Synthesis of results

Extracted data will be tabulated to aid with identification of commonalities and variations in the factors deemed important in the included studies. The tabulation will also aid with the identification of subgroups within the data. Characteristics of the included studies will be tabulated alongside the factors they identify as affecting access to healthcare. This will facilitate discussion of variations in what factors are important in different groups of countries based on social, economic and cultural characteristics. Variations in factors affecting access will be synthesised for subgroups of counties based on, for example, their income classification, their geographical location, the type of healthcare system, and cultural characteristics (subgroups identified will depend on the available data). Where subgroups are identified, synthesis will reflect on the commonalities across studies within the subgroup, and differences as compared with other studies/subgroups, as appropriate. A descriptive, narrative synthesis will be used to summarise the data from different studies, framed with reference to the strength and quality of the evidence. Findings of included reviews will be narratively described including explanation of their study characteristics with reference to the clinical area and study population on which they focus, the number and type of included study and conclusions drawn in terms of factors identified as influencing healthcare access. Influencing factors will be discussed in relation to the three delays model to examine where in the patient pathway it is common to find barriers/facilitators to healthcare access in different settings [9]. Where applicable, these factors will be broken down and explained as demand-side (patient) factors or supply-side (healthcare provider) factors. In the event that multiple studies have identical study setting and study design/focus but present different findings in terms of the factors influencing healthcare access, the results will be synthesised and discussed together as jointly presenting factors affecting access for that particular group.

## Discussion

While fair access to safe, affordable healthcare is internationally recognised as key to achieving the global goals, to date no study has considered factors affecting access to healthcare through a global lens. This study aims to address that gap by bringing together evidence of what factors affect patients’ ability to access healthcare and explore how those factors vary in different countries/contexts around the world. Factors affecting patients’ ability to access healthcare will be identified and analysed according to different country and context characteristics to shed light on the importance of different factors in different settings. The results may guide future research in this area and contribute to priority setting for development initiatives aimed at ensuring equitable access to healthcare for all. Any deviations from the methods outlined in this protocol will be documented and justified when results are presented.

There are a number of potential limitations that should be considered. Due to resource constraints, only articles published in English will be included in this overview. This could lead to evidence selection bias as studies published in other languages will be excluded from the pool of evidence collected. In addition, only published research will be eligible for inclusion. While we recognise this could introduce reporting bias into our findings, this was necessary to ensure enough information was available for data extraction and quality appraisal. Given that our research question is not clinical in nature, perhaps the risk of reporting bias is reduced as there is no ‘positive’ or ‘negative’ results as such, and instead the focus is on the real-life context and processes associated with receiving healthcare. There are also potential biases and quality issues in individual studies that feed into the reviews included in this overview. We also expect there to be a lack of weighting of individual studies given the likelihood of qualitative and mixed methods systematic reviews and consequently findings will be considered equally. A further limitation of this review is the large amount of relevant research which can cause difficulties in summarising and interpreting data. We have addressed this by adopting an overview of reviews approach to keep the review manageable and by taking a structured yet pragmatic approach to data extraction and synthesis. Nevertheless given the heterogeneity of the studies likely to be included, difficulties with synthesis of data and assessments of bias may remain.

One methodological difficulty in synthesising findings from overviews of systematic reviews is overlapping systematic reviews, i.e. if a study is included in more than one review which is included in the overview [11]. This problem is brought about by the layers of synthesis in an overview as compared to a systematic review. For overviews looking to combine results of systematic reviews quantitatively (e.g. in meta-analysis of treatment effect), there is possible bias if included systematic reviews contain the same papers. This can mean some studies are double counted in the analysis. As this review aims to synthesise evidence narratively, the effects of this type of bias are perhaps less significant. In addition, steps were taken in the design of the study to minimise this bias. For example, limiting the year of publication to a 5-year window is likely to minimise the number of repeated reviews in a particular area or setting. Nevertheless, reference lists of included studies will be examined to identify any studies that are included in multiple reviews. These will be identified explicitly within the narrative synthesis and accounted for in the interpretation.

As the focus of this review is to collect and examine factors (barriers and facilitators) affecting access to healthcare from existing systematic reviews, the decision was taken to exclude reviews synthesising evidence about only one barrier, facilitator or intervention. This decision was taken as reviews of this type may ignore other factors that might also be important and may introduce a bias towards factors that are pre-determined. However, we recognise that articles of this type may provide additional insights into factors affecting access to healthcare, how they are perceived and perhaps more detailed information about select barriers or facilitators. Consequently, although we will exclude this type of reviews from the main overview, these records will be grouped separately to enable additional analysis to be conducted after the main overview. A data extraction form will be developed and piloted for this purpose which will be informed by the main overview. This data extraction is likely to be more simplified than that for the main overview and the data will be synthesised narratively.

## Supplementary information

**Additional file 1.** PRISMA-P Checklist.

**Additional file 2.** Sample search strategy.

## Data Availability

N/A

## Abbreviations

AMSTAR: Assessing the methodological quality of systematic reviews
PRISMA: Preferred reporting items for systematic reviews and meta-analyses
