# Supplementary material for: What factors affect patients’ access to healthcare? Protocol for an overview of systematic reviews
Source: Syst Rev. 2020 Jan 23;9:18. doi: 10.1186/s13643-020-1278-z (PMC6979353; doi:10.1186/s13643-020-1278-z)
Supplement: Supplementary file 2 — Additional file 2. Sample search strategy. [file 13643_2020_1278_MOESM2_ESM.docx]

## Sample search strategy

1 Health Services Accessibility/

2 (availab* or unavailab* or access* or inaccessib* or used or using or usable or usage or "take up" or take-up or obtain* or utilis* or utiliz* or admit*).ti.

3 (((health or healthcare or nursing or medical or primary or secondary) adj2 (service* or system* or care or facillit* or centre or center)) or healthcare or hospital or pharmacy or pharmacies).ti.

4 adolescent health services/ or child health services/ or community health nursing/ or community mental health services/ or community pharmacy services/ or counseling/ or family planning services/ or hospices/ or maternal health services/ or occupational health services/ or dental health services/ or emergency medical dispatch/ or emergency service, hospital/ or emergency services, psychiatric/ or ambulances/ or health services for persons with disabilities/ or health services for the aged/ or health services for transgender persons/ or health services, indigenous/ or exp mental health services/ or exp nursing care/ or nursing service, hospital/ or ambulatory care/ or "bloodless medical and surgical procedures"/ or critical care/ or hospitalization/ or palliative care/ or perinatal care/ or perioperative care/ or perioperative period/ or preconception care/ or prenatal care/ or preoperative care/ or terminal care/ or pharmacy service, hospital/ or diagnostic services/ or immunization programs/ or vaccination coverage/ or rehabilitation/ or reproductive health services/ or exp rural health services/ or social work/ or suburban health services/ or urban health services/ or women's health services/ or emergency medical services/ or air ambulances/

5 exp ambulatory care facilities/ or birthing centers/ or dental clinics/ or health facilities, proprietary/ or exp hospitals, proprietary/ or hospital units/ or exp clinical observation units/ or exp hospitals/ or pharmacies/ or rehabilitation centers/

6 3 or 4 or 5

7 (barrier* or enabler* or motivat* or equalit* or inequalit* or facilitat* or obstruct* or limit* or imped* or hinder* or inhibit* or bottleneck* or deny* or denies or impact* or affect* or equit* or inequit*).ti.

8 1 and 7

9 2 and 6 and 7

10 8 or 9

11 exp animals/ not humans/

12 10 not 11

13 limit 12 to "systematic review"

14 (systematic adj3 review).tw.

15 12 and 14

16 13 or 15

17 limit 16 to yr="2014 -Current"
